# Melkerrson-Rosenthal Syndrome, a rare case report of chronic eyelid swelling

**DOI:** 10.1186/1746-1596-8-188

**Published:** 2013-11-13

**Authors:** Babita Kajal, John Harvey, Salem Alowami

**Affiliations:** 1 Department of pathology and molecular medicine, McMaster University, 1280 Main street west, Hamilton, Ontario L8N 3Z5, Canada; 2 Department of ophthalmology, McMaster University, 1280 Main street west, Hamilton, Ontario L8N 3Z5, Canada

**Keywords:** Melkerrson Rosenthal syndrome, Granulomatous disease

## Abstract

**Abstract:**

Melkerrson-Rosenthal syndrome is a rare disorder of unknown etiology. The classical triad of recurrent facial paralysis, swelling of the face, lips and deep furrowed tongue (Lingua Plicata) is seen in very few cases, majority of the patients often present with one or two symptoms only, which often leads to misdiagnosis and mismanagement. Clinically these symptoms vary from days to years, which further delay the definitive diagnosis and symptoms may eventually become permanent. The cause of this entity is not very well understood, but thought to be attributable to multiple entities including genetic and Infectitious. As this entity has been associated with numerous other clinical entities, diagnosis often remains an exclusion process. Methods: A middle age male with a chronic history of heavy eyelids with skin indurations predominately around left eye was presented to ophthalmology clinic. Physical examination revealed a deep furrowed tongue. The skin biopsy from left eyelid revealed a non-specific granulomatous lesion. The clinical correlation of facial swelling and deep plicated tongue prompted the differential of MRS Results: Histologically a non-specific granulomatous lesion was seen in dermis. As a rule, other causes of granulomatous diseases were ruled out especially Crohn’s disease and sarcoidosis. Polarization failed to reveal any foreign body. Conclusions: The finding of granulomatous lesion and clinical picture led to the definite diagnosis of Melkerrson-Rosenthal syndrome. Association with rosacea was other findings. Clinically his sign and symptoms are under control and no occurrence of symptoms has been noted so far.

**Virtual slides:**

The virtual slide(s) for this article can be found here: http://www.diagnosticpathology.diagnomx.eu/vs/1647494495993706

## Background

Melkerrson Rosenthal syndrome is a rare neurological disorder characteristically shows swelling of face, lips, facial paralysis and deep plicated tongue [1]. This classical triad is not seen in all of the patients, majority of the patients often show one or two features only. Duration of sign and symptoms also adds to clinical dilemma as these symptoms may vary from days to years, which eventually become chronic enough to cause permanent disfigurement of the facial part involved. Cheilitis granulomatous of Miescher is considered as monosymptomatic form of MRS. This entity has worldwide distribution and equally involves both genders. The definite etiology is still unknown. Despite the factor that numerous studies have been done to prove it as genetic or infectious entity, this remains a diagnosis of exclusion. Autosomal dominance has been proposed in few cases with chromosomal involvement of 9p11. Association of MRS has been seen with many other clinical entitles. Histological examination reveals a nonspecific granulomatous lesion with dermal edema and perivascular lymphocytic infiltrate. As in any granulomatous lesions, differential list is very exhaustive. Crohn’s and sarcoidosis needs to be ruled out first, as these diseases may co express or may develop during the course of MRS [2,3]. Histologically granulomas may not been seen in all cases, but that does not rule out the diagnosis of MRS. The granulomatous reaction and clinical findings of eyelids swelling, deep furrowed tongue often helps in the definite diagnosis of Melkerrson Rosenthal syndrome. Patient may respond to steroids and other immunosuppressants. Clinically high suspicion index is required to solve this clinical quandary.

## Case report

**Figure 1:**
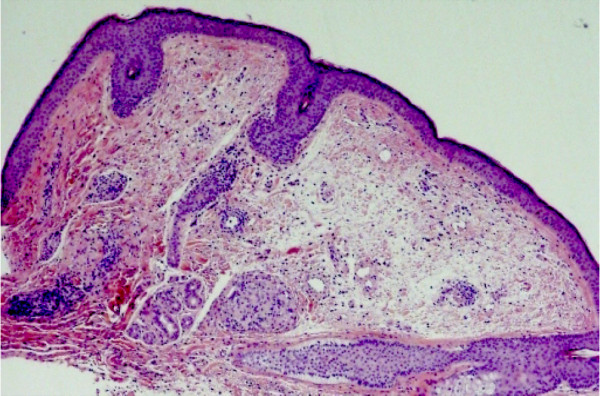
Dermal edema, peri-lymphatic infiltrate and noncaseating granuloma.

**Figure 2:**
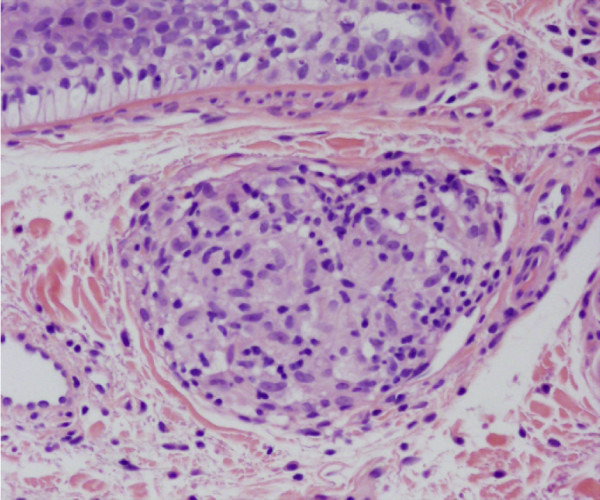
Non caseating granuloma.

**Figure 3:**
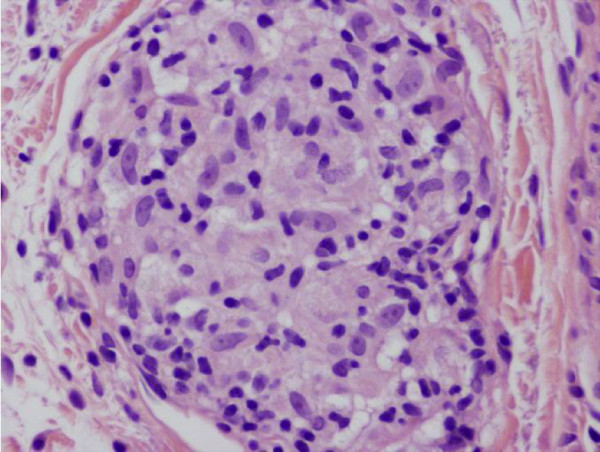
Higher power, non caseating granuloma.

**Figure 4:**
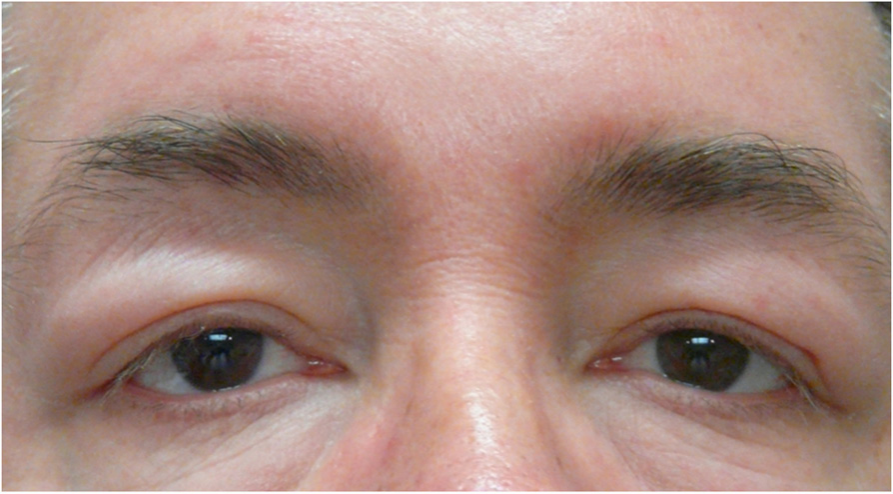
Eye lid swelling.

**Figure 5:**
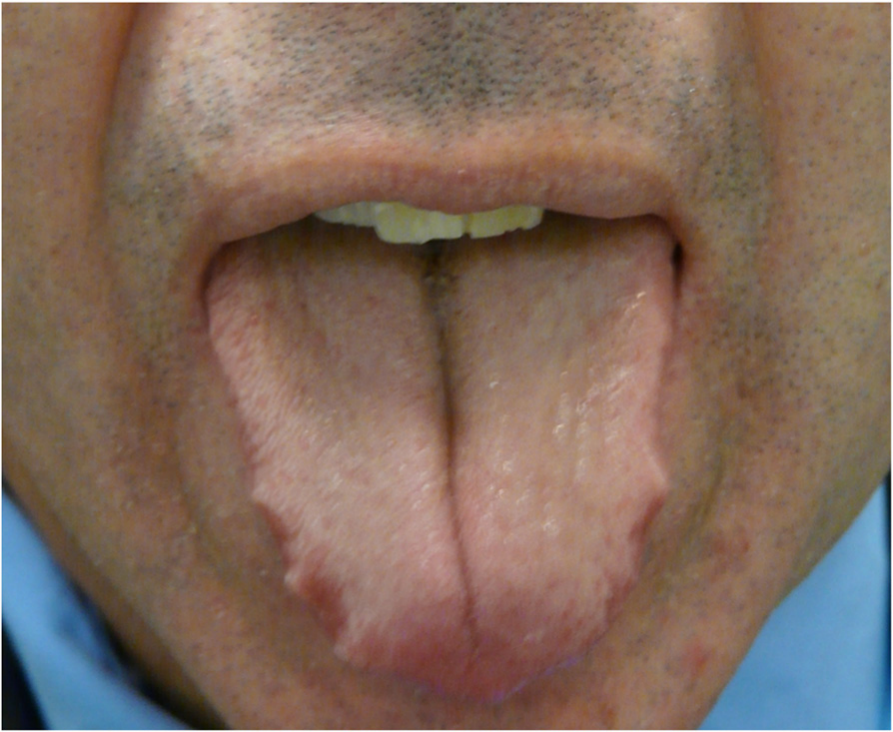
Deep plicated tongue.

A 51-year-old male presented with clinical symptoms of bilateral heavy lids, predominantly on left side of one-year duration. No itchiness, no redness was found. Working clinical diagnosis of allergy, facial rosacea, or blepharocalasis was made. A 3 mm punch biopsy taken from left lid swelling and submitted for histopathological examination. His past medical history include hernia repair, left knee scope for benign cyst, left shoulder repair. Family history was remarkable for colon cancer, but colonoscopy was normal for patient. No other significant medical history given in chart. In our department we received a left eye lesion skin biopsy, measuring 0.3x0.3x0.3 cm, submitted in one block. Microscopic sections revealed unremarkable epidermis with mild degree of hyperkeratosis. Underlying dermis showed edema, perivascular lymphocytic infiltrate and well defined perilymphatic non- necrotizing granulomas. Descriptive diagnosis of “edema with mild chronic inflammation and focal perilymphatic granulomas” was given (Figures 1, 2, 3). Fungal, bacterial and mycobacterium stains were negative. Polarization failed to reveal foreign body. The diagnosis of MRS was suggested based upon chronic eyelids swelling and deep plicated tongue (Figures 4 and 5). The serology was negative for c ANCA, p ANCA, angiotensin converting enzyme, and cyclic citrullinated peptide antibody. Patient was started on steroid therapy and doxycycline, patient responded excellent for both eyelid swelling and rosacea.

## Discussion

Melkerrson Rosenthal syndrome is a rare neurological entity of unknown etiology. Classical triad includes recurrent facial palsy, swelling of lips, and deep furrowed tongue (lingua plicata, sometimes known as scrotal tongue, but these classical findings are seen rarely [4]. Majority of the patient has one or two symptoms only rather than classical triad as mentioned above. MRS has considerable overlap or rather confusing terminology, especially when classical triad is not seen. Miescher cheilitis may be a monosymptomatic form of the Melkerrson-Rosenthal syndrome, but probably these are two separate entities. Approximately 86% of the cases present with orofacial swelling and furrowed tongue are almost present congenitally [5]. Fissured and woody tongue may also be seen in other conditions including lipoid proteinosis, which is a rare autosomal recessive disorder characterized by deposition of PAS positive material in various organ systems and is caused by loss of function mutation in gene encoding ECM1 (extracellular matrix protein 1) on chromosome 1q21 [6]. In available literature, there is no ECM 1 protein abnormality seen in MRS. The term MRS used only when cheilitis occurs with facial palsy and deep furrowed tongue. The term orofacial granulomatosis (OFG) is used when there is no underlying Crohn, s disease. This entity is seen equally in both gender and has worldwide distribution. Due to inconsistency of sign and symptoms appearance and duration of symptoms, majority of patient’s goes undiagnosed for long period of time, as long as for 30 years, and may eventually become permanent [7]. Recurrent swelling of eyes and upper lip usually involves the same side. The facial palsy is notably seen in the region of VIIth nerve distribution, which can be mistaken clinically as Bells palsy. Severity of palsy also may vary from mild to severe, which could be unilateral or bilateral. Involvement of other cranial nerves also has been noted such as olfactory auditory; glossopharyngeal and hypoglossal nerves [8]. Numbness can involve other body parts also.

Regarding pathogenesis this is not a well understood entity, multiple attempts to prove this entity as inherited/ acquired/ neurological or immunological has yield no conclusive data. Many authors associate this syndrome with some form of herpes infection, other consider as an immunological response to multiple circulating antigen which leads to allergic granulomatous reaction. Many factors are thought to attribute to etiology namely genetics and infections particularity oral candidiasis and mycobacterium. Other etiologies under consideration are autoimmune factors, hypersensitivity and neurotrophic factors, which leads to vasomotor disturbances causing numbness. Deficiency of complement C1-INH may be the etiological factor contributing to orofacial swelling [9]. DNA sequence study of MAP1S900 (Mycobacterium avium subspecies paratuberculosis (MAP) has been found in few studies [10,11]. In another case study, attempt to find borrelia burgdorferi a spirochete as an etiological agent did not reveal any positive results [12].

An autosomal dominant inheritance with variable expression has been proposed in some cases of MRS and de-novo translocation of t (9; 21) (p11; p11) has seen in many cases [13]. Seven family members of four generations have been described forming the genetic basis of this entity [14]. Attempt to study the blood DNA for UNC-93B1 gene mutations predisposing to herpes virus infection in patients of MRS with facial palsy yield no results [15]. Is this a clonal disorder of T lymphocytes remains to be determined? [16]. So far there are many speculations about the pathogenesis, but without any definite conclusions.

MRS with facial palsy of Melkerrson syndrome may present alone or as a disease spectrum of various other underlying diseases, such as association has been seen in many other chronic diseases including diabetic mellitus [17], Crohn’s disease [18]. Ehler –Danlos syndrome [19], Psoriasis [20], Crocodile Tear syndrome [21], Rosacea [22], Hashimoto thyroditis [23], Leprosy [24] and Down syndrome [25]. Hereditary Melkerrson Rosenthal syndrome and multiple sclerosis has been noted in one family [26]. The term MRS is used only when there is no other underlying etiology.

A retrospective diagnosis of total 72 biopsied cases was done at mayo clinic and concluded it as a chronic disease that may present over the course of most of the lifespan and may require several years of observation to be diagnosed. Neurologists who observe a combination of facial edema and facial palsy in a patient should consider the diagnosis of MRS and proceed to a diagnostic skin biopsy and a trial of steroid treatment for their patient [27]. Melkerrson-Rosenthal syndrome should be suspected in cases of recurrent facial palsy associated with swelling of orofacial structures and furrowed tongue [28].

Histologically non-caseating granulomas are seen in dermis. Other findings included perivascular inflammatory infiltrate and dermal edema. The granulomas may be seen around lymphatic and blood vessels [29]. Few of the cases might not show any granulomas, but lymphangiectasia and perivascular lymphocytic infiltrate only. This is a diagnosis of exclusion as extensive laboratory and clinical work up is required to rule in and rule out various other immunological or non-immunological conditions.

Differential diagnosis of MRS includes thyroid retinopathy, allergy, atophy, angioedema, bacterial, viral or filarial infections, systemic lupus erythematosus, dermatomyositis, bell’s palsy, leprosy and rosacea. Non-caseating granulomas in biopsies is a nonspecific finding which further requires a detailed workup.

As MRS is a chronic disorder of relapses and remissions, main emphasis of treatment is symptomatic to relieve the swelling and pressure symptoms. Many treatment options include steroids, immunosuppressant therapy, and reconstructive surgery [30,31]. In follow-up care, one must exclude the development or coexistence of Crohn’s disease or sarcoidosis.

## Conclusions

MRS is a disease spectrum which might co express with many other clinical entities. Our emphasis is on the skilful and detailed workup between treating physician in solving the diagnostic quandary of this rare and confusing entity. We conclude that Melkerrson - Rosenthal syndrome should in the differential diagnosis of orofacial swelling and fissured tongue. Our patient responded well to prednisone and doxycycline [32].

## Consent

Written informed consent was obtained from the patient for the publication of this report and any accompanying images.

## Abbreviations

MRS: Melkerrson- Rosenthal Syndrome.

## Competing interests

The authors declare that they have no competing interests.

## Authors’ contributions

BK drafted the manuscript and collected data; SA analyzed histopathological sections and correlated with clinical findings. JH provided the clinical details; written consent and treatment follow up of the patient. All authors read and approved the final manuscript.
